# Mitofusin-2 Independent Juxtaposition of Endoplasmic Reticulum and Mitochondria: An Ultrastructural Study

**DOI:** 10.1371/journal.pone.0046293

**Published:** 2012-09-28

**Authors:** Pierre Cosson, Anna Marchetti, Mariella Ravazzola, Lelio Orci

**Affiliations:** Department for Cell Physiology and Metabolism, Centre Médical Universitaire, Geneva Faculty of Medicine, Geneva, Switzerland; Ecole Polytechnique Federale de Lausanne, Switzerland

## Abstract

Besides its role in controlling the morphology of mitochondria, mitofusin-2 has been proposed to tether mitochondria to the endoplasmic reticulum (ER), based largely on light microscopic analysis. In this study we have examined by electron microscopy the organization of ER and mitochondria in cells expressing or not mitofusin-2. Contrary to previous studies, we observed that loss of mitofusin-2 increased ER-mitochondria juxtaposition. These results suggest that mitofusin-2 does not play a critical role in the juxtapostion of ER and mitochondria, and highlight the essential role of ultrastructural analysis to visualize and measure contact between two intracellular compartments.

## Introduction

In eukaryotic cells, the endoplasmic reticulum (ER) is the starting point of the secretory pathway where secreted proteins are co-translationally inserted, folded, modified and sorted [1]. ER also plays a key role in controlling intracellular calcium homeostasis, firstly as the major intracellular store of calcium, and secondly by controlling calcium influx through the plasma membrane [2]. This regulatory function is achieved by establishing an intimate contact between the ER and the plasma membrane [3], allowing proteins in the two membranes to interact directly (reviewed in [4]). The ER can also associate closely with mitochondria [5]. In mammalian cells it has long been recognized that calcium released from the ER is transferred very efficiently to mitochondria, probably because the close proximity of the two organelles allows high local concentrations to be achieved in the cytosol [6]. Close contact between the ER and mitochondrial membranes also allows direct transfer of phospholipids between these two organelles [7]. Cross-talk between mitochondria and ER is essential for cell survival in yeast [7], and plays a critical role in the control of cell death in mammalian cells [8].

Molecular mechanisms controlling juxtaposition of ER and mitochondria, usually referred to as mitochondria-ER tethering mechanisms, have been a subject of active investigation in recent years (reviewed in [9]). Genetic analysis in yeast identified the key role of the ERMES complex [7]. It is not clear however if an equivalent complex exists in mammalian cells. On the other hand, at least two other ER-mitochondria tethering mechanisms have been proposed in mammalian cells. Firstly, Grp75 has been observed to form a complex with mitochondrial VDAC and ER IP3 receptor [10]. Although the original study was focused on the functional role of this complex, this interaction has occasionally been cited as a potential mechanism for anchoring the ER to mitochondria [8]. Secondly, it has been reported that loss of mitofusin-2 altered the ER morphology, and reduced strongly contacts between ER and mitochondria, suggesting a role for mitofusin-2 in tethering ER to mitochondria [11]. Mitofusin-2 was extensively characterized as a mitochondrial protein controlling tethering and fusion of mitochondria with each other [12], [13], although it was suggested in this later study that a small fraction of mitofusin-2 may be present in the ER [8], [11]. Mutations in mitofusin-2 are the main cause of Charcot-Marie-Tooth neuropathy type IIa [14], and a better knowledge of the function of this protein is essential to understand the etiology of this disease.

Ultrastructural analysis by electron microscopy is a powerful tool to study relationships between cellular compartments. It provides a finer resolution than light microscopy and can thus allow the detection of qualitative changes in the morphology of cellular structures. Morphometric analysis also provides a rigorous quantitative evaluation of cellular structures. Contacts between the ER and mitochondria can be observed at the ultrastructural level by electron microscopy, both in chemically fixed and in cryopreserved samples [15]. These observations have revealed a close apposition (within 10–20 nm) of the mitochondrial outer membrane and the ER membrane, and the establishment of protein tethers between the two membranes [5], [15], [16], [17].

In this study, we have examined at the ultrastructural level the role of mitofusin-2 in ER-mitochondria juxtaposition by comparing wild-type (WT) and mitofusin-2 (*mfn2*) KO cells. Our aim was to examine the morphology of these two cellular compartments, and to evaluate qualitative and quantitative changes induced by the loss of mitofusin-2.

## Results and Discussion

### Increased ER-mitochondria juxtaposition in *mfn2* KO cells

**Figure 1:**
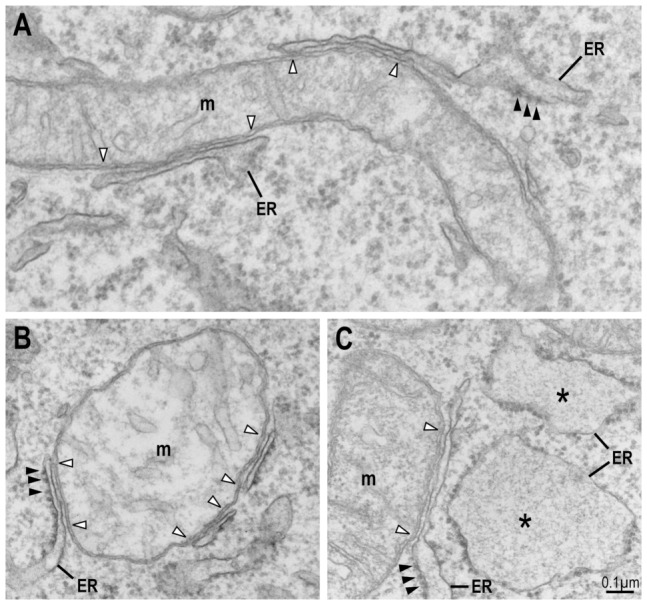
Visualization of ER-mitochondria tethering by electron microscopy. Cells were fixed, embedded in Epon resin, sectioned and observed. (A) In WT MEFs expressing mitofusin-2, compartments continuous with ER cisternae were closely apposed to the mitochondrial outer membrane. Full arrowheads point to ribosomes attached to ER cisternae. Empty arrowheads designate the limits of the zone of intimate contact between ER and mitochondria. m: mitochondria. (B, C) Close ER-mitochondria apposition was also observed in *mfn2* KO cells. In these cells, ER cisternae often appeared swollen (asterisks), but regions of tethering were still very thin.

In order to determine the role of mitofusin-2, we examined at the ultrastructural level the morphology of mitochondria and ER, with particular attention to the regions of close apposition between them. As previously observed in other cell types [5], [15], [16], [17], in mouse embryonic fibroblasts (MEF) ER and mitochondria engaged into close contacts that were visualized by electron microscopy in fixed cells (Fig. 1A, empty arrowheads). The size and morphology of these contact areas was very variable. Segments of the ER engaged into contact with mitochondria often appeared thinner than ER cisternae and the membrane of the ER facing the mitochondria was deprived of ribosomes (Fig. 1A).

We quantified the relative abundance of ER-mitochondria contacts in three independent experiments and determined that 2.25% of the mitochondrial membrane was engaged into close contact (<20 nm) with the ER (Table 1). In *mfn2* KO cells [11], we observed a similar organization and structure of mitochondria-ER contacts (Fig. 1B–C). Surprisingly, in these cells a much higher percentage of the mitochondrial membrane (4.91%) established close contacts with the ER (Table 1). This difference was not due to an increase in the average size of the contacts, which was essentially similar in the two situations, but rather to an increase in their frequency (Table 1).

**Table 1:** ER-mitochondria contacts are increased in *mfn2* KO cells.

	Mitochondria profiles		Contacts with ER			
Experiment	number	membrane length (µm)	number	membrane length (µm)	size (nm)	% total membrane
1-WT	254	539	92	10.7	116	1.99
1-*mfn2* KO	110	301	136	17.1	126	5.67
2-WT	310	633	110	15.3	139	2.41
2-*mfn2* KO	249	658	213	29.8	140	4.53
3-WT	82	158	34	3.92	115	2.47
3-*mfn2* KO	92	208	90	10.4	116	4.97
Total						
WT	646	1330	236	29.9	127	2.25
*mfn2* KO	451	1167	439	57.3	131	4.91

Since the frequency of mitochondria-ER contacts was calculated here relative to mitochondrial membranes, we then verified if the absolute amounts of ER and mitochondria were modified in *mfn2* KO MEFs relative to WT cells. For this we used 80 random pictures (20 µm^2^ each) from four independent experiments. For each picture we determined the surface occupied by the ER and mitochondria in order to estimate the volume of the cell occupied by these compartments. We also measured in each picture the length of membrane delimiting these compartments, to obtain an estimate of the surface of the two compartments (Table 2). Only relatively small differences were observed between the two cell types, notably a small but significant increase in the amount of ER membrane in *mfn2* KO cells(+27%). In *mfn2* KO cells, we also detected a small increase in the volume occupied by mitochondria (+25%), while the amount of membrane of this compartment did not increase (Table 2). The cell volume, determined by electric current exclusion, was also similar for WT (2957fl±172; n = 3) and *mfn2* KO cells (2551fl±86; n = 3). Together these results indicate that mitochondria-ER contacts are approximately two times more abundant in *mfn2* KO cells than in WT cells.

**Table 2:** No significant alteration in the overall surface of mitochondria in *mfn2* KO cells.

	Wild-type	*mfn2* KO	p value (t-test)
Total area (µm2)	1607	1548	
ER area (%)	5.84±0.66	5.99±0.61	>0.05
ER membrane (µm/µm2)	1.29±0.08	1.64±0.08	<0.01
mitochondria area (%)	8.95±0.51	11.2±0.77	0.012
mitochondria membrane (µm/µm2)	0.75±0.04	0.73±0.04	>0.05

### Mitofusin-2 controls ER morphology

In agreement with previous reports [11], we also observed that ER cisternae often appeared more dilated in *mfn2* KO cells than in WT cells (Fig. 1C, asterisks), although a wide variety of ER profiles was apparent. In order to quantify the morphology of the ER, we measured in three independent experiments the surface and the length of 50 ER profiles, to calculate the average width of individual cisternae. Since regions of the ER engaged in close contact with mitochondria were often very thin even in *mfn2* KO cells (see Fig. 1C), these regions were not taken into account in this quantification. In each experiment, cisternae appeared significantly wider in *mfn2* KO than in WT cells (Table 3). A very high variability was observed from experiment to experiment, suggesting that small variations in the procedure (e.g. cell culture or fixation) had a strong influence on the morphology of the ER. These results are in general agreement with previous studies, which revealed that the morphology of mitochondria and the ER is affected in *mfn2* KO cells [11], [12], [13].

**Table 3:** ER cisternae are dilated in *mfn2* KO cells.

	ER profiles		Student t-test
Experiment	number	width (nm) ±SEM	
1-WT	52	78.9±7.69	0.024
1-*mfn2* KO	49	101.4±6.27	
2-WT	51	53.8±3.14	<0.001
2-*mfn2* KO	52	178.4±15.1	
3-WT	56	117.6±16.2	0.012
3-*mfn2* KO	55	156.9±16.3	

### Complementation of *mfn2* KO cells

**Figure 2:**
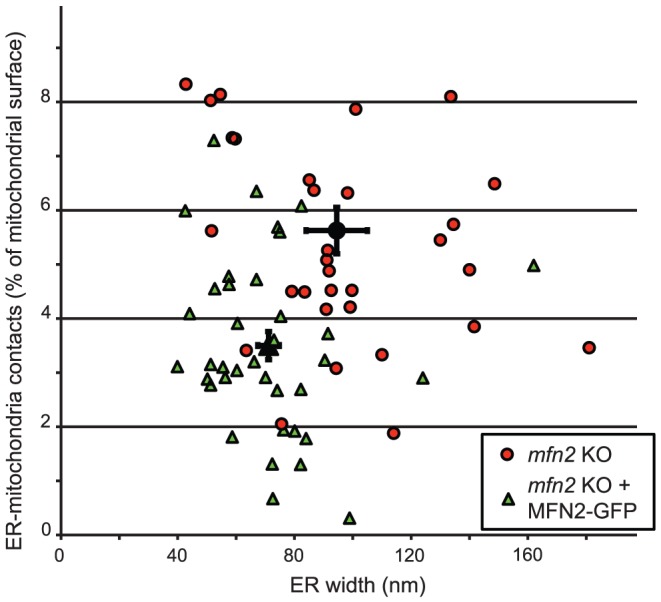
Complementation of mitofusin-2 KO cells. Mfn2 KO cells were transfected with a plasmid driving the expression of Mitofusin-2 fused to GFP. Cells expressing moderate levels of fluorescence were sorted, re-grown, fixed and processed as described in the legend to Fig. 1. For each individual cell profile, the frequency of ER-mitochondria tethering (expressed as % of the total mitochondrial perimeter) was determined, as well as the average width of ER cisternae. Mock-transfected *mfn2* KO cells (red circles) exhibited wider ER cisternae and more ER-mitochondria tethering than cells re-expressing Mitofusin-2 (green triangles). For each population of cells, the average and S.E.M. are indicated in black.

Embryonic fibroblasts are obtained from very early stages of development. Differences between two distinct cell lines (e.g. WT and *mfn2* KO) may be partially caused or masked by differences in the exact differentiation stage of each cell line. In order to evaluate directly if the differences observed between WT and *mfn2* KO cells were caused by the lack of mitofusin-2, we complemented KO cells with a plasmid expressing mitofusin-2 fused to the green fluorescent protein (GFP). Transiently transfected cells were sorted by FACS to select cells expressing a moderate level of fusion protein, then fixed and processed for ultrastructural analysis. As a control, cells were transfected with an empty vector and submitted to the same procedure. As reported previously [12], [18], overexpression of mitofusin-2 resulted in massive intermitochondrial adhesion and clustering, and disrupted mitochondrial morphology at the ultrastrucutral level. This phenotype was observed in approximately 10% of sorted cells, and these cells were excluded from further analysis. For each condition, the morphology of the ER as well as the abundance of mitochondria-ER contacts was analyzed in at least a dozen individual cell profiles (Fig. 2). In two independent experiments, expression of mitofusin-2-GFP in *mfn2* KO cells reduced significantly the width of ER cisternae, as well as the number of ER-mitochondria contacts (Fig. 2 and Table 4).

**Table 4:** Expression of Mfn2-GFP complements the phenotype of *mfn2* KO cells.

Complementing plasmid	Number of cell profiles	ER width (nm)	ER-mitochondria contacts (%)
Exp 1			
1-pRC	11	117±10	4.84±0.55
1-Mfn2-GFP	13	73.4±4.1	2.55±0.29
p value (t-test)		<0.01	<0.01
Exp 2			
2-pRC	20	79.7±5.4	6.29±0.58
2-Mfn2-GFP	25	70±5.2	3.99±0.33
p value (t-test)		0.20	<0.01
Total			
1-pRC	31	94.5±5.83	5.62±0.43
2-Mfn2-GFP	38	71.1±3.7	3.5±0.26
p value (t-test)		<0.01	<0.01

**Figure 3:**
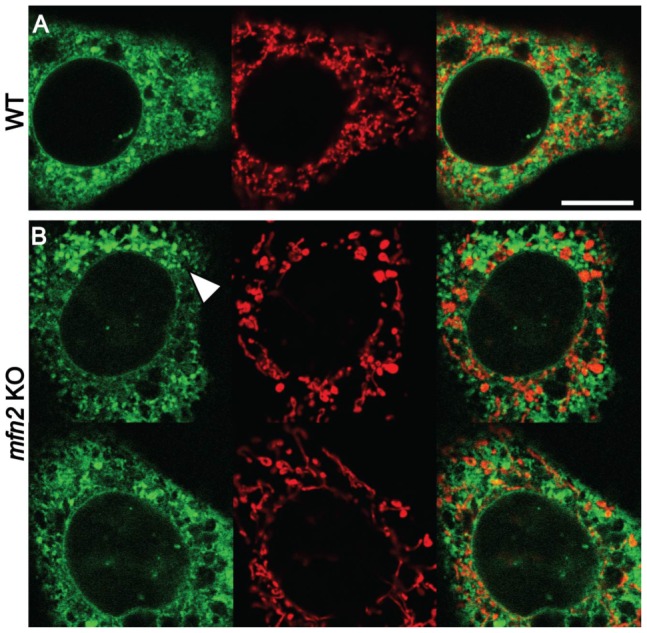
Fluorescence analysis of ER and mitochondria. WT (A) and *mfn2* KO cells (B) were co-transfected with a GFP-coupled ER marker (green) and an RFP-coupled mitochondrial marker (red). After fixation, confocal images were acquired and used to quantify Manders' colocalization coefficients (Table 5). The arrowhead indicates a region of a *mfn2* KO cell where the ER appears dilated. Bar: 10 µm.

Our observations are difficult to reconcile with previous observations suggesting that loss of mitofusin-2 decreased significantly ER-mitochondria tethering [11]. Our results indicate that the juxtaposition of the two organelles was actually significantly increased in *mfn2* KO cells compared to WT cells. In agreement with this observation, mitochondria-ER contacts decreased upon re-expression of mitofusin-2 in *mfn2* KO cells. To ascertain that this discrepancy was not due to variations in experimental procedures, we first verified that mitofusin-2 was expressed in WT MEFs, and not in the *mfn2* KO cells used in this study (data not shown). We then reproduced previously reported fluorescence experiments [11] and in four independent experiments we also observed that Manders' B colocalization coefficient was reduced in *mfn2* KO cells compared to WT cells (Fig. 3 and Table 5). It thus seems likely that the apparent discrepancy between our study and previously published results mostly reflects the different methods used to quantify ER-mitochondria juxtaposition, and in particular the difference in resolution between electron and light microscopy.

**Table 5:** Manders' colocalization coefficients in WT and *mfn2* KO MEF cells.

	Manders A			Manders B		
Experiment	WT	*mfn2* KO	p-value	WT	*mfn2* KO	p-value
1	0.88+/−0.01 (n = 19)	0.92+/−0.01 (n = 18)	<0.05	0.59+/−0.02 (n = 19)	0.48+/−0.02 (n = 18)	<0.01
2	0.87+/−0.01 (n = 27)	0.90+/−0.01 (n = 20)	>0.05	0.67+/−0.02 (n = 27)	0.60+/−0.02 (n = 20)	= 0.01
3	0.93+/−0.01 (n = 25)	0.96+/−0.01 (n = 25)	<0.05	0.62+/−0.02 (n = 25)	0.51+/−0.02 (n = 25)	<0.01
4	0.91+/−0.01 (n = 24)	0.93+/−0.01 (n = 24)	>0.05	0.67+/−0.02 (n = 24)	0.48+/−0.02 (n = 24)	<0.01

Classical fluorescence microscopy localizes cellular structures with a resolution of at best 200 nm, while electron microcopy provides a resolution of 1 nm, which is essential to ascertain direct contact between two membranes (i.e. a distance shorter than 20 nm). In principle, based on classical fluorescence pictures, one may only draw conclusions regarding the proximity of two organelles rather than their propensity to establish direct contacts. The limitations of light microscopy are confounded when attempting to calculate the degree of colocalization of two compartments. Various coefficients can be used to this effect, such as Manders' coefficients [19], but their interpretation is subject to severe limitations [20], [21].

Besides general considerations, in the situation analyzed here, a few specific parameters may complicate the evaluation of colocalization by fluorescence microscopy. First, since regions of the ER juxtaposed to mitochondria often appear very thin by electron microscopy, they may be particularly difficult to visualize by fluorescence microscopy when volumetric probes (e.g. BiP, or GFP fused to a KDEL ER localization signal) are used to localize the ER. Second, the morphology of both the ER and mitochondria is altered by loss of mitofusin-2, and it is difficult to evaluate how such changes may affect the apparent degree of colocalization as measured by fluorescence. Third, the Manders' B coefficient used to measure colocalization of ER and mitochondria [11] effectively measures the percentage of ER engaged into contact with mitochondria. Since our results suggest that the total amount of ER membrane may be increased in *mfn2* KO cells, this may cause an apparent decrease in the percentage of ER contacting mitochondria. One or several of these factors may contribute to an apparent decrease in colocalization, as measured by fluorescence microscopy. Based on these considerations, use of electron microscopy appears more adequate than fluorescence microscopy to measure juxtaposition of ER to mitochondria.

In summary, our observations indicate that the juxtaposition of ER and mitochondria is increased by loss of mitofusin-2, suggesting that mitofusin-2 is not essential for this process, and actually inhibits it. This does not contradict the fact that the functionality of mitochondria-ER contacts is affected in *mfn2* KO cells, as suggested by the previous observation that calcium transfer from the ER to mitochondria is altered in these cells [11]. More refined studies will be essential to understand how mutations in mitofusin-2 result in Charcot-Marie-Tooth neuropathy [14]. By revealing the possible discrepancies between observations made by light and by electron microscopy, this work strongly suggests that studies of the role of mitofusin-2, and more generally studies of the propensity of cellular compartments to engage into direct contact, should include extensive observations by electron microscopy coupled to morphometric analysis.

## Methods

### Cell culture and reagents

MEF cells were described previously [11], [22], and grown at 37°C in Dulbecco's modified Eagle's medium (DMEM, Gibco), supplemented with 10% fetal calf serum (FCS, Gibco) and non-essential aminoacids (Sigma). For transfection, cells were seeded at day 1 in a 100 mm-diameter tissue culture dish, to reach about 80% confluency at day 2. Transfection was performed at day 2 with 15 µl of Lipofectamine 2000 (Invitrogen) and 6 µg of plasmid DNA, in 10 ml of serum-free medium. The following day, the medium was replaced with fresh DMEM containing FCS. At day 4, cells were detached with versene (Gibco), resuspended in PBS (Gibco) at a concentration of 10^6^ cells/ml, and sorted by FACS (FacsVantage SE, BD). Cells expressing medium levels of mitofusin-2-GFP were selected and transferred into a 35 mm culture dish. At day 5, cells were fixed and processed for electron microscopy.

To determine cell volume, cells were detached with versene, washed once in PBS and resuspended at 1^.^10^4^ cells/ml before analysis with a Casy 1 counter (Schärfe System).

Colocalization of mitochondria and ER was measured essentially as described previously [11]: WT and *mfn2* KO cells were co-transfected with MT-RFP and ER-GFP plasmid DNA using Lipofectamine 2000 (Invitrogen). 24 hours after transfection, cells were fixed with 4% paraformaldehyde and observed in a LSM510 confocal microscope (Zeiss). Pictures were acquired and Mander's A and B colocalization coefficients were calculated with Imaris® software.

### Electron microscopy

For conventional electron microscopy, cells grown in 35 mm plastic dishes were fixed with 2% glutaraldehyde buffered with 0.1 M sodium phosphate, pH 7.4, postfixed with osmium tetroxide, stained with uranyl acetate [23], dehydrated in ethanol and embedded in Epon. After sectioning, the samples were observed in a Tecnai electron microscope (FEI). The AnaLysis software was used for quantification.

### Western blot

The equivalent of 40 µg of protein per lane (29^.^10^4^ cells) was migrated on a 9% acrylamide gel and transferred on a nitrocellulose membrane (Whatman). The nitrocellulose was incubated with an anti-mitofusin-2 mouse antibody (Abnova), then with an anti-mouse-HRP-coupled andibody (Biorad), and finally revealed by ECL.
